# Ocular indicators of Alzheimer’s: exploring disease in the retina

**DOI:** 10.1007/s00401-016-1613-6

**Published:** 2016-09-19

**Authors:** Nadav J. Hart, Yosef Koronyo, Keith L. Black, Maya Koronyo-Hamaoui

**Affiliations:** 1 Department of Neurosurgery, Maxine Dunitz Neurosurgical Research Institute, Cedars-Sinai Medical Center, 127 S. San Vicente Blvd., Los Angeles, 90048 CA USA; 2 Department of Biomedical Sciences, Cedars-Sinai Medical Center, 110 George Burns Rd., Los Angeles, CA 90048 USA

**Keywords:** Alzheimer’s disease, Amyloid-beta, Tauopathy, Retinal biomarkers, Ocular abnormalities, Neurodegenerative disease

## Abstract

Although historically perceived as a disorder confined to the brain, our understanding of Alzheimer’s disease (AD) has expanded to include extra-cerebral manifestation, with mounting evidence of abnormalities in the eye. Among ocular tissues, the retina, a developmental outgrowth of the brain, is marked by an array of pathologies in patients suffering from AD, including nerve fiber layer thinning, degeneration of retinal ganglion cells, and changes to vascular parameters. While the hallmark pathological signs of AD, amyloid β-protein (Aβ) plaques and neurofibrillary tangles (NFT) comprising hyperphosphorylated tau (pTau) protein, have long been described in the brain, identification of these characteristic biomarkers in the retina has only recently been reported. In particular, Aβ deposits were discovered in post-mortem retinas of advanced and early stage cases of AD, in stark contrast to non-AD controls. Subsequent studies have reported elevated Aβ_42/40_ peptides, morphologically diverse Aβ plaques, and pTau in the retina. In line with the above findings, animal model studies have reported retinal Aβ deposits and tauopathy, often correlated with local inflammation, retinal ganglion cell degeneration, and functional deficits. This review highlights the converging evidence that AD manifests in the eye, especially in the retina, which can be imaged directly and non-invasively. Visual dysfunction in AD patients, traditionally attributed to well-documented cerebral pathology, can now be reexamined as a direct outcome of retinal abnormalities. As we continue to study the disease in the brain, the emerging field of ocular AD warrants further investigation of how the retina may faithfully reflect the neurological disease. Indeed, detection of retinal AD pathology, particularly the early presenting amyloid biomarkers, using advanced high-resolution imaging techniques may allow large-scale screening and monitoring of at-risk populations.

## Introduction

Since Alzheimer’s disease (AD)-type senile dementia was first described in 1905 by Alois Alzheimer, great efforts have been made to better understand its manifestation in the brain [67, 76]. The disease is characterized by a spectrum of cognitive and neuropsychiatric symptoms, including severe memory loss, behavioral changes, disorientation, visual impairments, sleep disturbances, and, at late stages: difficulties walking, swallowing and, invariably, death [88, 153]. Classical AD neuropathology involves the accumulation of misfolded endogenous proteins, hallmarked as extracellular amyloid β-protein (Aβ) plaques, and intracellular neurofibrillary tangles (NFT), which result from the aggregation of hyperphosphorylated tau protein (pTau) [11, 66, 76, 153, 165, 166]. These insidious pathologies can arise decades before substantial neurodegeneration and brain atrophy. Unfortunately, by the time symptoms suggestive of clinical diagnosis appear, damage may be too extensive for effective intervention [88]. A century following its first report, AD and associated dementia are estimated to afflict 47 million people worldwide, a number projected to triple by 2050 [15]. This age-dependent epidemic is a major concern for the aging population, with an incidence that rises sharply after 65 years of age, affecting roughly 50 % of individuals aged 85 and older [51].

Clinical, genetic, physiologic, and biochemical evidence suggest that the primary and earliest pathological event leading to AD is the accumulation of Aβ in the brain, which appears as a net result of imbalance between production and clearance [76, 88, 127, 153, 166]. Amyloid β-protein precursor (AβPP), a large transmembrane protein, undergoes multiple cleavage events to generate Aβ peptides [76, 165]. According to the prevalent amyloid hypothesis of AD, the disease-associated amyloidogenic pathway involves cleavage by both β-secretase and γ-secretase to produce the longer, aggregation-prone Aβ_40_ and Aβ_42_ alloforms [76, 85, 165]. Aβ_42_, which is more specifically associated with AD, may exert its neurotoxic and cognitively detrimental effects through an array of conformational structures, ranging from small, soluble oligomers to insoluble fibrils that often culminate in degenerating neurites, termed senile (neuritic) plaques [76, 156, 165, 166]. Our expanding knowledge of tauopathy in AD brains, including intracellular tangles and threads of aggregated pTau, has grown to encompass a diversity of extracellular soluble and insoluble assemblies, which may induce seed-like self-propagation into synaptically dense regions [11, 33, 66, 77, 84, 94, 182]. Currently, the detection of plaques and NFTs by histological brain examination at autopsy provides the most definitive diagnosis of AD [4, 55, 76, 81, 88, 95, 166]. Although plaque burden may plateau at a presymptomatic stage of the disease, obscuring its relationship with disease progression, it is postulated that the early assemblies of misfolded Aβ also elicit chronic, low-grade neuroinflammation that correlates with cognitive decline [27, 131, 153, 169, 186].

Modern brain-imaging techniques, such as magnetic resonance imaging, that detect cerebral atrophy or measure functional metabolic changes are instrumental in differentiating healthy aging from pathological conditions [29, 43, 54]. However, these tissue alterations are common to many neurodegenerative disorders, and thus cannot be used to unequivocally distinguish AD from other types of dementia [96, 153]. Advanced positron emission tomography (PET) brain imaging of hallmark amyloid and tau AD pathology using various radioactive tracers (e.g., ^11^C-Pittsburgh compound B, PiB [125], ^18^F-florbetapir [45], ^18^F-flutemetamol [80], ^18^F-florbetaben [173], ^18^F-TKH5105, and ^18^F-T807 [89]) provides disease specificity and facilitates ongoing research. However, it may be difficult to deploy this technology for population-wide screening of preclinical signs due to high cost, necessity of using radioactive isotopes, limited resolution, and the resulting unfeasibility of longitudinal studies [144]. Should screening become possible, early stage intervention at the level of Aβ aggregation, pTau, synaptic dysfunction, and inflammation may allow clinicians to modulate disease progression. To better serve the population at risk for developing AD, new methods of definitive and non-invasive diagnosis are needed.

With various reports of retinal structural deficits, other ocular abnormalities, and even visual dysfunctions experienced by AD patients [19–22, 31, 35, 59, 60, 69, 81, 90, 97, 98, 107, 108, 113, 130, 163, 180], it is no surprise that the field has begun shifting its attention to the eye as a site of AD manifestation. The retina is a CNS tissue originating in the developing diencephalon, and it contains high-density neuronal cells and fibers that form a sensory extension of the brain [25]. It also shares many structural and functional features with the brain, including the presence of neurons, glial cells, a blood barrier, and similar cell-fate specification of embryonically related tissues as well as tight regulation of endothelial cell proliferation [25, 126, 176]. Furthermore, axons of the optic nerve connect the retina to the brain directly and facilitate the transportation of AβPP synthesized in RGCs in small transport vesicles [136].

The first evidence of nerve degeneration in the human AD eye was reported by Hinton et al., in 1986 [81]. Since then, the reports of retinal pathology in patients with AD have grown to include RGC loss, NFL atrophy, thinning of the macular ganglion cell complex, and widespread axonal degeneration in the optic nerve [17, 19, 21, 22, 38, 73, 81, 92, 97, 147, 174]. Other changes, such as blood flow rate [19, 50, 59, 183], signs of inflammation [20, 21], and varied cellular degeneration mirroring those observed in the AD brain may reflect cerebral pathology [21, 76, 128, 186], but do not indicate AD as strongly as disease hallmarks. However, the subsequent identification of retinal Aβ plaque pathology was specific to AD patients and early stage cases, and matched amyloid pathology in the brain [107]. This was further validated by other independent studies on AD patients [1, 113, 178] that parallel findings in animal models of the disease. The latter, predominantly involving transgenic (Tg) rodents, reported similar retinal patterns, where Aβ deposits often colocalize with sites of apoptosis, neuroinflammation, impairments of function and structure, and plaque formation that even precedes that seen in the brain [46, 107, 108, 139, 148, 149, 152].

As the only CNS tissue not shielded by bone, the retina offers unique access for direct and non-invasive imaging to study possible pathological changes in the brain. Moreover, since recent studies suggest that other diseases, such as multiple sclerosis, ischemic stroke, and Parkinson’s disease, also exhibit retinal abnormalities similar to the cerebral pathologies observed, the retina represents an appealing target to detect neurodegenerative disease [7, 26, 143]. The evidence of Aβ accumulation in early stage cases and amyloid-related neurodegeneration in the AD retina [107, 113] may support its status as a site of presymptomatic stage imaging, and even suggests that Alzheimer's is both a cerebral and an ocular disease. This review provides an updated report of ocular hallmark pathologies and other abnormalities observed in patients and animal models of AD, as well as methods used to detect these changes in vivo and to monitor them in response to therapeutic intervention.

## Hallmark pathology in ocular tissues of AD patients

As established cerebral hallmarks of AD, Aβ and pTau protein aggregates most strongly indicate the occurrence of the disease [76, 77, 159, 165, 166]. Nearly a century following the first description of these signs in the brain, documentation of their manifestation in ocular tissues has begun to emerge [1, 69, 91, 100, 107, 113, 163, 178]. Table 1 presents key findings from research on hallmark AD pathology in the ocular tissues of human patients.

**Table 1:** Abnormalities observed in ocular tissues of AD patients

Tissue		Pathology	Findings	References
CNS				
Retina		Amyloid	Elevated AβPP expression	[1, 69]
			Increased Aβ_40_ and Aβ_42_ peptide levels	[1]
			Classical and diffuse extracellular Aβ plaques, plaque-like structures, and intracellular Aβ deposits, particularly in the superior quadrant and innermost layers	[100, 107, 113, 178]
			Aβ deposits inside and around degenerating melanopsin (m)RGCs^a^	[113]
		Tau	pTau positive in GCL, IPL, INL, ONL, and OPL	[91, 163]
		Neuronal degeneration	NFL thinning and macular volume loss in all quadrants; Mostly in superior and inferior quadrants; Diffuse dropout; NFL thickness correlated with MMSE^b^ score, NFL abnormalities	[10, 14, 19, 20, 34, 50, 79, 87, 91, 104, 106, 110, 113, 114, 118, 121, 124, 135, 137, 142, 145–147, 160, 167, 177]
			GCL degeneration from mild to severe; RGC swelling; Loss of mRGCs and dendritic arborization	[20, 113, 160]
		Inflammation	Extensive cell processes suggesting gliosis from ILM^c^ adjacent to GCL	[20]
		Vascular	Perivascular Aβ deposition	[113]
			Reduction of venous blood flow, column diameter, speed, arteriolar and venular fractal dimensions, branching complexity and geometric optimality; Changes to arteriolar and venular tortuosity; Increased width variation; Blood flow correlated with NFL thickness	[19, 32, 50, 59, 183]
			Elevated oxygen saturation	[49]
		Other	*In vivo* FLIO^d^ imaging: Auto-fluorescence changes correlated with MMSE score and CSF pTau	[91]
Optic nerve		Structural	Large caliber fiber loss; Superior and nasal quadrant axonal loss, overall reduction; Cup-to-disc ratio increase; Intraocular pressure susceptibility increased; Optic disc paleness; Suggested link to glaucoma	[81, 113, 160] [14, 16, 40, 81, 121, 171, 177]
Non-CNS				
Lens		Amyloid	Elevated AβPP expression	[69]
		Other	Cytosolic electron-dense Aβ nanodeposits in supranuclear, cortical regions and in anterior epithelial subregion in DS^e^ lens	[69, 101, 133]
			Supranuclear cataracts; Opacity^f^; PS1^g^ expressed in DS lens	[18, 49, 57]
Cornea		Other	PS1 expressed in the DS cornea	[57]
Aq. humor		Amyloid	Increased Aβ_40_ levels	[69]
Choroid		Structural	Thickness reduction	[63, 178]

^a^Retinal ganglion cell

^b^Mini-Mental State Examination

^c^Inner limiting membrane

^d^Fluorescence lifetime imaging ophthalmoscopy

^e^Down’s syndrome

^f^Opacity difference not statistically significant

^g^Presenilin-1 protein

**Fig. 1:**
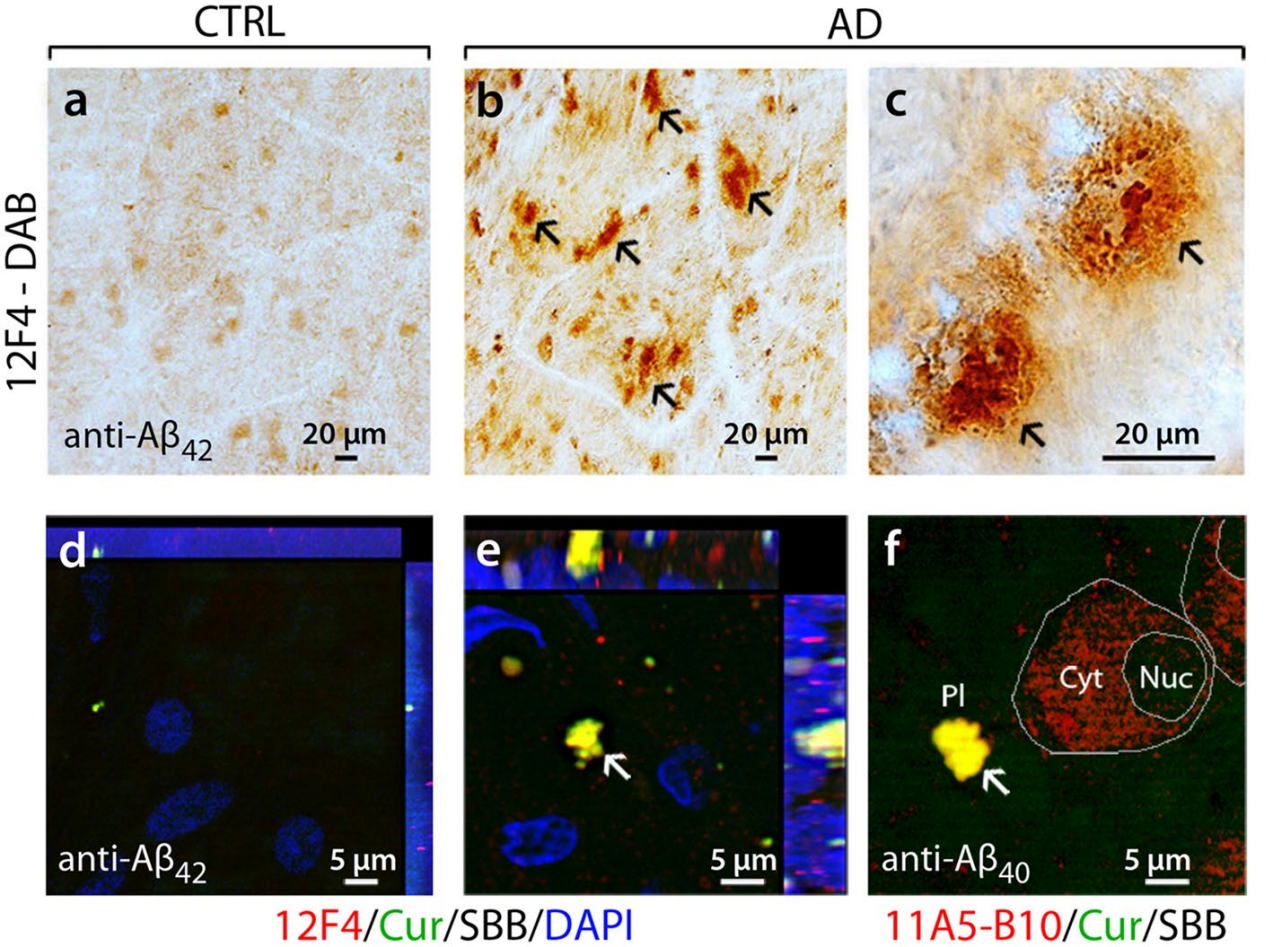
Flat-mount retinas from AD patients exhibit the accumulation of Aβ deposits. **a–c** Representative microscopic images from a definite AD patient (74 years) and a matched control individual (CTRL; 71 years) stained with anti-Aβ_42_ C-terminal-specific antibody (12F4) and visualized with peroxidase-based labeling (DAB). Blood vessel structures seen as lighter lanes. **c** Classical mature Aβ plaques observed along a retinal blood vessel. **d**, **e** Fluoresence labeling of Aβ_42_-containing deposits detected in retina of AD patient (*yellow*), using curcumin (*green*), 12F4 antibody (*red*), and DAPI nuclear staining (*blue*). Sudan Black B (SBB) is used to quench non-specific autofluorescent signal. **f** Compact extracellular Aβ plaque and cytosolic Aβ_40_ accumulations observed following curcumin and anti-Aβ_40_ C-terminal-specific antibody (11A5-B10) staining in post-mortem retinas of AD patients. *Arrows* indicate various types of Aβ plaques Images **a**-**c** adopted from La Morgia et al., Annals of Neurology, vol. 79, no. 1, pp. 90–109, 2015

Before amyloid-related aggregation was shown in the AD-afflicted eye, Aβ immunoreactivity in the sub-retinal pigment epithelium (RPE) was reported in normal aged eyes by Loeffler et al. [119]. While the study did not examine eyes from AD patients, Goldstein et al. [69] reported the detection of Aβ nanoaggregates in the human AD lens, a non-CNS tissue. Notably though, it was not until 2010 that Koronyo-Hamaoui and colleagues (2011) were able to demonstrate the existence of extra-cerebral Aβ deposits in the human AD retina, a CNS tissue [107]. Sequence-specific monoclonal antibodies and amyloid conformation-detecting compounds (i.e., curcumin and thioflavin-S) were used to identify Aβ deposits in flat-mount retinas from definite AD patients and suspected early stage cases [107]. In this study, AD diagnosis was determined by pre-mortem cognitive assessment and a detailed neuropathological report assessing existence of cerebral neuritic plaques, neurofibrillary tangles, neuropil threads, and amyloid angiopathy; age- and gender-matched non-AD controls did not meet these criteria. Subsequent studies revealed that retinal plaques possibly associate with blood vessels in the superior quadrant and exhibit a morphological array similar to amyloid pathology observed in the brain [107, 113, 178]. In retinas from human patients, Aβ deposits with single or multiple compact globular cores appeared more frequently than classical plaques with central Aβ cores and radiating fibrils [107]. Examples of extracellular Aβ plaques, containing Aβ_40_ and Aβ_42_ alloforms, and intracellular Aβ_40_ immunoreactivity detected by several labeling techniques in retinas of AD patients in contrast to age-matched controls are shown in Fig. 1; unpublished data and data reported in La Morgia and colleagues [113].

Shortly thereafter, a study by Alexandrov et al. [1] that used both biochemical methods and histological examination of post-mortem eyes provided evidence for increased Aβ peptide levels, particularly Aβ_42_, in retinas from AD patients (Table 1). In addition, AβPP immunoreactivity was elevated in AD retinas, justifying the expected elevation of Aβ_40_ and Aβ_42_ peptides, as well as the resulting formation of amyloid plaques [1]. In 2014, Aβ plaque-like structures morphologically denser than those observed in a Tg rat brain in the same study were described in two retinas from AD patients [178]. Later that year, in vivo detection of amyloid deposits in AD retinas using a method of guided optical coherence tomography (OCT) was reported. Findings included mostly perimacular and perivascular spots in the outer plexiform layer (OPL), ganglion cell layer (GCL), and NFL [100].

**Fig. 2:**
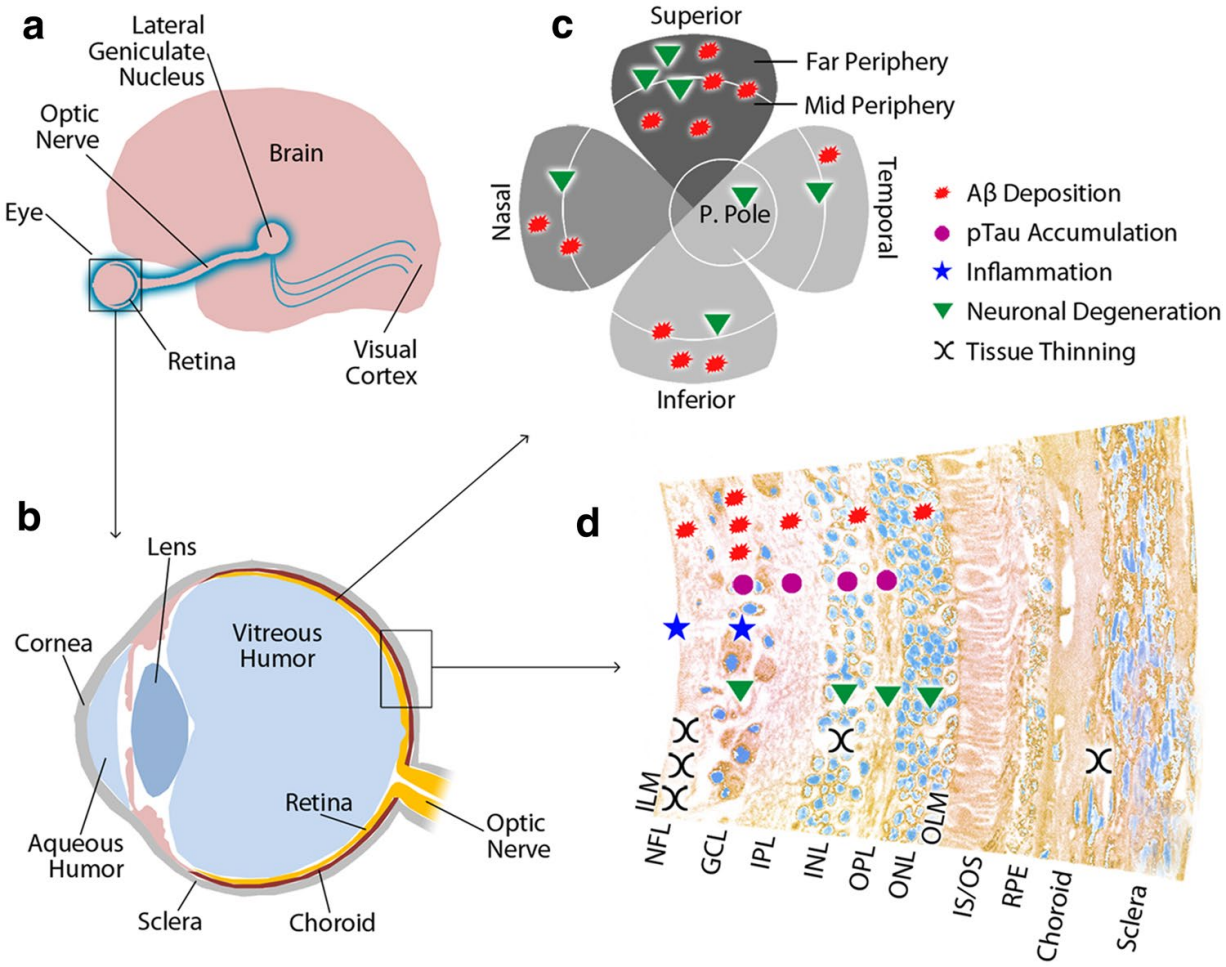
Manifestations of AD in the Human Retina. **a** Visual pathway. **b** Eye-sagittal plane. **c** Retinal flat-mount shows the geometric distribution of pathology by quadrant with more consistent findings of NFL thinning indicated by darker shading. **d** Cross section of retina and adjacent ocular tissues shows the distribution of pathology by tissue layer. *Aβ* amyloid beta-protein, *pTau* phosphorylated tau, *NFL* nerve fiber layer, *GCL* ganglion cell layer, *IPL* inner plexiform layer, *INL* inner nuclear layer, *OPL* outer plexiform layer, *ONL* outer nuclear layer, *ILM* inner limiting membrane, *OLM* outer limiting membrane, *IS/OS* inner and outer segments of photoreceptor layer, *RPE* retinal pigment epithelium, *P. Pole* posterior pole

More recently, La Morgia and colleagues (2016) further demonstrated the appearance of classical and morphologically diverse Aβ aggregates, which often appear in clusters in retinal flat-mounts from definite AD patients. Importantly, this study was the first to report the accumulation of Aβ deposits in and around degenerating melanopsin retinal ganglion cells (mRGC), further suggesting that Aβ is toxic to retinal cells. Colocalized Aβ immunoreactivity was also detected in degenerating neurites of mRGCs [113]. Figure 2 illustrates AD-related ocular findings in the human eye, with an emphasis on the retina. In addition to the above findings, the evidence of the neurotoxicity of Aβ to retinal cells has been shown in various investigations. Cell-line studies have demonstrated Aβ-induced RGC cell death and RPE senescence [28, 179]. Animal model studies have shown RGC apoptosis accompanied by and colocalizing with Aβ deposits in retinas from rodent models of AD or glaucoma, while the reduction of Aβ levels by immunization led to the structural preservation of the RPE and visual protection in a murine model of Age-related Macular Degeneration (AMD), suggesting that Aβ causes neurodegeneration in these models [44, 74, 139]. Furthermore, a study reported that retinal Aβ injection induced photoreceptor degeneration in a wild type (WT) mouse, and that exposing RPE cells to Aβ in vitro reduced mitochondrial redox potential and production of reactive oxygen species [24].

In addition to growing reports of retinal Aβ accumulation, one study has reported hallmark pTau in the retina, while another has reported indirect indication of pTau [91, 163]. A notable study by Schön et al. [163] provided the first evidence of pTau in retinal cross sections of AD patients, particularly in the innermost layers (Fig. 2), although the group was unable to detect fibrillar Tau and Aβ aggregates. Tau hyperphosphorylation was detected by anti-AT8 immunoreactivity, which binds phosphorylated groups at Ser202 and Thr205 [163]. A subsequent study showed significant evidence that changes in retinal fluorescent lifetime imaging ophthalmoscopy correlate with total Tau and pTau-181 concentration in the cerebrospinal fluid (CSF) [91]. On the other hand, a study employing the standard staining protocols for brain tissues, was unable to detect aggregates of Aβ, pTau, or α-synuclein upon examination of retinal cross sections and other ocular tissues from AD and Parkinson’s disease (PD) patients [82]. It is important to note, however, that studies from eight independent groups examining eyes from AD patients have consistently found retinal tauopathy and increased formation of Aβ deposits in retinas and lenses [1, 69, 101, 102, 107, 113, 133, 163, 178], and two recent studies examining retinas from patients with PD or Tg mice modeling PD and Dementia with Lewy Bodies reported the presence of misfolded α-synuclein within the inner retinal layers, along with impaired vision [23, 155].

Although outnumbered by evidence of changes in the neurosensory retina, a CNS tissue, some studies have reported the detection of Aβ peptides and nanoaggregrates in non-CNS ocular tissues. These include the description of amyloid-related changes to the human AD and Down’s Syndrome (DS) lens, including AβPP immunoreactivity and Aβ nanoaggregation in supranuclear, cortical, and anterior epithelial subregions of the lens [69, 101, 133]. Individuals with DS, in which chromosome 21 trisomy results in triple copies of the AβPP gene, are at increased risk for early onset AD, and as such offer valuable data pertaining to the disease [185]. Interestingly, one of these studies reported that Aβ burden in the lens of a 2-year-old DS individual was comparable with those observed in a 57-year-old familial AD patient and an 85-year-old sporadic AD patient [133]. In a recent study, in vivo detection of Aβ was reported in the lens, with a technique later used to successfully predict clinical AD diagnoses [101, 102]. Another report of non-CNS Aβ accumulation describes detection in the aqueous humor [69]. In contrast to the above findings, one study specifically reported no Aβ immunoreactivity in lenses and corneas from post-mortem AD donors [132]. While the accessibility of the lens has inspired enthusiastic study and subsequent reports of characteristic AD pathology, one must consider the degree to which lenticular and other non-CNS protein aggregates can accurately reflect cerebral amyloid burden during disease progression and in response to therapy.

As discussed above, recent studies have shown that certain tissues of the eye, particularly specific regions of the retina, present an abundance of characteristic AD pathology. Among these, parameters of amyloid aggregation coupled with RGC degeneration in the superior quadrant of the innermost retinal layers (i.e., NFL and GCL) might distinguish ocular pathology specific to AD from that observed in other neurodegenerative diseases, such as AMD and glaucoma [92, 93, 116, 120]. The evidence of key AD biomarkers Aβ and pTau in the neuroretina urges further exploration of Alzheimer’s in this ocular tissue.

## Non-specific ocular abnormalities in AD patients

In addition to hallmark biomarkers, the complex pathology of AD manifests as an array of ocular abnormalities, many of which have been repeatedly observed in AD patients. In this section, we explore cellular, structural, vascular, and other changes that may be associated with increased neurotoxic Aβ in the AD-affected eye (Table 1).

Pathological changes in the AD eye were first documented in 1986, when Hinton and colleagues reported optic nerve degeneration, a decrease in ganglion cell numbers, and thinning of the NFL [81]. Further reports of GCL pathology described the severe degeneration of RGCs, including a phenotype of vacuolated mitochondria and nerve fiber cell degeneration. The evidence of AD-related GCL degeneration has been accumulating since then, including a report of 25 % neuronal loss in the GCL, with the greatest losses noted in the superior and inferior retinal quadrants [20–22, 38]. Interestingly, two of these studies reported that while GCL loss is age-dependent in control retinas, it does not correlate with age in the AD eye [22, 38]. Sadun and colleagues [160] reported a loss of the largest caliber fibers in the optic nerve and degeneration of RGCs. Further studies of the AD eye have followed, reporting NFL and macular thinning, as well as optic nerve degeneration. Findings of NFL thinning have indicated a significant reduction of thickness among quadrants of this tissue layer [10, 13, 19, 34, 62, 87, 104, 106, 113, 114, 124, 135, 145–147, 167], although some findings did not reach statistical significance [1, 50, 79, 91, 110, 111, 167]. A small number of studies have provided evidence against NFL thinning. Two of these studies reported no change in NFL thickness compared with controls [91, 103]. In another study, age was found to be the greatest factor contributing to NFL thickness, while AD patients showed no significant thinning in comparison to controls [111]. Although reports of NFL thinning vary, there appears to be an overwhelming majority of evidence supporting a significant thinning of the superior quadrant of the NFL in the AD retina (Fig. 2) [19, 62, 79, 87, 104, 110, 113, 118, 121, 137, 142, 145–147, 160, 177].

Interestingly, NFL thinning has been correlated with an abnormal pattern electroretinography (pERG) response [146]. More recently, two studies reported a correlation between degree of cognitive impairment and either NFL thinning or macular volume reduction [87, 142]. Other findings have indicated that AD patients and control subjects show no significant differences when comparing the latency of visual evoked potential (VEP) P100 component [87], and no correlation between NFL thinning and Mini-Mental State Examination (MMSE) score [62]. Data showing NFL thinning and *macula lutea* reduction in AD seem to mirror results from studies on MCI [62, 104, 145]. While degeneration has been observed most notably in the NFL and GCL, it has also been observed in the inner nuclear layer (INL) [178]. It appears that the innermost layers of the retina show greater and more significant thinning than the outer retinal layers [17] (Fig. 2). A recent meta-analysis of NFL thinning in AD patients, including many of the above-described studies, reported that, after correction for varied methodology, the field shows consensus for a significant mean thinning of the NFL in AD [34]. While research strongly suggests that NFL measurement may be useful for the early diagnosis and evaluation of disease progression [87], further study is needed to optimize the utility of this method as a specific ocular biomarker of the disease. More recently, in a clinical study on AD patients exhibiting circadian abnormalities, La Morgia and colleagues (2016) described NFL thinning in the superior quadrant, measured by OCT [113]. More importantly, this study described degeneration specific to a photoreceptor subtype, mRGC, which makes up 1–2 % of all RGCs [112, 113]. Degenerating mRGCs were associated with Aβ deposits within and around mRGCs in post-mortem retinas from AD patients [113]. The mRGCs are known to modulate circadian photoentrainment by projecting to the hypothalamus suprachiasmatic nucleus (SCN), the circadian pacemaker of the brain [112]. This study may provide the first mechanistic explanation for the circadian dysfunction often reported in AD patients [113].

Since 2007, numerous findings of retinal angiopathy and other related vascular changes have also been documented in AD patients. These changes include narrowed veins, reduction of blood flow, vascular attenuation, increased width variation, reduction of branching complexity and optimality, smaller fractal dimensions, and changes in tortuosity [19, 32, 50, 59, 183]. A recent study by Einarsdottir et al. [49] reported that although vascular diameter was not changed, blood oxygen saturation was notably elevated in the AD retina with oximetry data closely matching that seen in AMD.

An interesting abnormality observed in the retina relates to the abnormal expression of members of the synuclein family of proteins (α-, β-, and γ-synuclein), with a retinal layer occurrence in AD patients distinct from that seen in healthy individuals [170].

One aspect of ocular pathology that has been repeatedly documented in AD patients is a significant elevation of cup-to-disc ratio, with one study reporting a threefold increase [16, 40, 121, 171, 177]. Interestingly, since cup-to-disc ratio is used to measure the progression of glaucoma [3], a number of investigations have explored the potential connection between glaucoma and AD. Two of these studies examined AD patients and found a relationship with one reporting that 25.9 % of the sample was positive for glaucoma, a condition that is only prevalent in about 5 % of the general population [16, 171]. Conversely, two recent studies that examined the risk of glaucoma patients developing AD found no relationship [12, 105]. Recently, optic disc color pallor, indicative of axon loss, has been investigated as a potential biomarker, given that the optic discs of AD patients show a significant paleness compared with those of controls [13]. In light of overlap between AD and glaucoma, some researchers have even termed glaucoma “Ocular AD” [16, 129]. However, apparent differences between glaucoma and the ocular manifestations of AD challenge such a notion. While glaucoma shows optic disc cupping accompanied by a specific pattern of loss in the optic nerve and GCL [3, 41], the ocular findings in AD have overwhelmingly indicated damage to the NFL and GCL along with hallmark molecular signs in peripheral retinal regions [20–22, 81, 107, 113, 163]. Since evidence linking the two diseases remains controversial, further research would be warranted before claims of common etiology could be made about these diseases.

Although most research on ocular degeneration in AD has focused on CNS tissues like the retina, a number of studies have reported changes in lenses of the AD and DS eyes. Reports have indicated supranuclear and deep cortical cataracts, opacity due to increased light scattering, and other changes that may be associated with the findings of Aβ peptides in the lens [49, 69, 101, 102, 133]. On the other hand, a study that examined amyloidopathy in cortical cataracts was unable to detect Aβ in lenses of patients with and without AD [132]. Studies examining changes in the AD eye have also reported significant thinning of the choroid [17, 63, 178].

## Visual dysfunction in AD patients and animal models

Among the earliest symptoms documented in some AD patients are visual impairments, especially loss of contrast and color sensitivity, limited visual field, compromised visual attention, reduced stereopsis, deficits in the perception of shape from motion, and impaired object and face recognition [9, 31, 90, 97, 138, 140, 161, 162, 174]. However, unlike well-established retinal structural deficits, various changes in visual function appear to manifest inconsistently across AD patients, and further study should expand upon the currently limited findings.

While many aspects of visual acuity, such as recognition, localization, and target detection, were not found to be significantly different in AD patients when compared with controls [31, 90], loss of contrast sensitivity was markedly different [64, 90, 157]. Since the latter parameter may be detected clinically in a routine eye examination, it may serve as a biomarker for AD-related neurodegeneration [31]. However, loss of color sensitivity and the possibility of using this parameter to determine AD status remain highly controversial [9, 31]. Abnormal visual field has also been correlated with disease severity, with AD patients showing sensitivity loss greater than controls [31, 90, 175]. Deficits in motion perception have been associated with severity of dementia, and evidence suggests that AD patients may have impaired motion sensitivity due to selective damage to the magnocellular pathway [31, 65, 83, 99]. Although reduced depth perception and stereopsis have been linked with cognitive impairment, opinions regarding the effect of stereopsis in AD patients are mixed [31, 115, 158]. Finally, saccadic eye movement is among the most well-described deficits to ocular motor function in AD patients [36, 53]. Nevertheless, related visual dysfunction, such as pupil size and pupillary light reflexes are not exclusive to AD and may also be found in healthy older individuals and in other neurological conditions such as PD [71].

Thus far, other functional changes detected through electrophysiology techniques have been limited but offer unexpected clues to the roots of visual abnormalities arising from neuroretinal dysfunction. Delayed pace of processing, measured by responsiveness of pERG, has been documented in AD patients [146, 147]. A study examining pattern VEP in AD patients found no significant difference between the AD and non-AD groups and no correlation with MMSE score [87]. Additional reports of AD-related electrophysiology deficits have come from animal model studies, showing abnormal flash VEP measurements in APP_SWE_/PS1_ΔE9_ mice [61]. Two studies reporting ERG measurements in 5xFAD and APP_SWE_/PS1_ΔE9_ mice found that the response was not significantly different between Tg and WT groups, but was instead correlated with age [150, 152].

While data regarding visual dysfunction have not been definitive, recent findings show a promising connection between circadian rhythm and retinal neuronal degeneration. La Morgia and colleagues (2016) reported that a subgroup of AD patients suffers from significantly reduced sleep efficiency due to circadian rhythm disruption that may be caused by RGC loss and Aβ toxicity in the retina [113]. This study diverges from the historical attribution of visual and ocular-related dysfunctions in AD patients to brain abnormalities [112, 113].

Although most of these changes are modest in magnitude, taken together they may impact daily activity and cognitive performance significantly. These studies suggest that visual abnormalities in AD, historically attributed to brain pathology, may arise directly from pathology in the retina, such as Aβ- and tau-derived neurodegeneration, optic nerve atrophy, inflammation, and vascular attenuation. As we continue to explore potential treatment, growing evidence of the ocular aspects of AD suggests that therapeutic intervention should address visual as well as cognitive dysfunctions. Mitigation of deficits, such as contrast insensitivity, delayed pace of processing, and retinally regulated circadian functions, could meaningfully improve quality-of-life for those suffering from this debilitating disease.

## Ocular findings shared by AD and AMD

Certain similarities between AD and AMD have drawn attention to a potential connection between these degenerative conditions. AMD is an ocular disease characterized by sub-RPE drusen deposits, thickening of Bruch’s membrane, and degeneration of the RPE and photoreceptors within the *macula centralis* [2]. Like AD, the risk of developing AMD increases exponentially with age [58, 141]. As AMD progresses, patients show central visual field loss [2, 141], which differs from the inferior visual field loss described in some AD patients [175]. The primary degeneration noted in AMD typically takes place in the photoreceptors of the macula and in the underlying RPE, while AD retinas primarily show degeneration in the GCL and NFL [10, 13, 19, 21, 22, 34, 62, 81, 87, 104, 106, 113, 114, 124, 135, 145–147, 167]. Interestingly, Aβ has been detected in the eyes of both AMD and AD patients, within drusen deposits in AMD [42, 122] and as the primary constituent of extracellular fibrillar plaques in AD retinas [107, 113]. In the report by Dentchev et al. [42], Aβ was detected in drusen deposits in the retinas of 4 out of 9 AMD patients, but not in drusen deposits from normal eyes. Another study examining drusen deposits did not find amyloid fibrils within drusen, but identified amyloidogenic oligomers, suggesting that Aβ oligomers may be involved in the biogenesis of drusen deposits [122]. Interestingly, in an earlier study by Loeffler et al. [119], patchy Aβ immunoreactivity was detected in sub-RPE deposits in eyes from normal older persons but not in retinas from patients with AMD. These deposits corresponded to either soft drusen or basal linear deposits [119]. Other abnormalities common to AMD [156, 168] have also been observed in the eyes of AD patients, including abnormal retinal blood circulation [19], vascular changes [58, 141], reduced NFL thickness [145], foveal RGC degeneration [20], and choroidal thinning [63].

In the 5xFAD Tg mouse model, RPE degeneration, which is also characteristic of AMD, has been reported in two recent studies [148, 150], one of which even found drusen-like deposits and Bruch’s membrane thickening [148].

Multiple genetic studies on these diseases have revealed a tight connection to the ApoE gene. However, while the associated risk of sporadic AD increases with the ApoE4 allele and decreases with the ApoE2 allele, the opposite has been observed in AMD (reviewed in [168]). Shared components of the complement system, a part of innate immunity, suggest that common inflammatory mechanisms are involved in AD and AMD. In addition, the oxidative stress experienced by photoreceptors of AMD patients is mirrored by the AD brain as increased reactive oxygen species, oxidative damage, and mitochondrial dysfunction [168]. Taken together, the findings of AD and AMD commonalities suggest a degree of overlap, yet, key differences in retinal layer and geometric distribution of hallmark pathologies warrant further investigation towards defining differential diagnosis.

## AD-specific ocular pathology in animal models

Recent work on animal models of AD has shed light on the biological role of AβPP, soluble Aβ peptides, insoluble Aβ aggregates, and pTau species in the eye. Advances include the identification of the species and aggregates that may interfere with essential cellular mechanisms at early stages of the disease. This section summarizes the current findings of characteristic AD abnormalities in the eyes of rodent and fly models of the disease (Table 2).

**Table 2:** AD-specific ocular biomarkers in animal models

AD pathology	Species	Model	Findings	References
Amyloid-related				
AβPP expression	Drosophila	AβPP^a^/dBACE^b^/dPsn^c^	Expression in fly eye; Over-expression in all murine retinal layers, optic nerve, cornea, lens	[46, 47, 56, 72, 117, 139, 188]
	Mouse	Tg2576^d^, APP/PS1^e^		
	*O. degus* ^f^	Sporadic AD (aged)		
Aβ peptides	Drosophila	pGMR-Aβ_42_^g^, dBACE-AβPPL^h^	Increased levels in retinal extracts from fly; NFL, GCL, IPL, INL, OPL, OS^i^, RPE (extra- and intracellular), choroid, cornea, lens, vitreous humor, including oligomeric Aβ species and Aβ engulfed by astrocytes; In *O. degus*, more peptides and oligomers detected in central retina	[1, 30, 39, 46, 48, 52, 72, 75, 107, 117, 134, 139, 148, 150, 154, 184]
	Mouse	Tg2576, APP/PS1, 3xTg^j^, 5xFAD^k^		
	*O. degus*	Sporadic AD (aged)		
Aβ deposits	Drosophila	AβPP, dBACE-AβPPL, pGMR-Aβ_42_	Retinal extracts from fly; Deposits and/or plaques in most retinal layers (strongest in GCL and central retinal region), optic nerve, sclera; Endogenous dAβ^l^ deposits	[1, 30, 46–48, 72, 75, 107, 117, 139, 152, 178, 187]
	Mouse	Tg2576, APP/PS1, 3xTg, 5xFAD		
	*O. degus*	Sporadic AD (aged)		
	Rat	TgF344-AD^m^		
Vascular Aβ	Mouse	Tg2576, APP/PS1	Vascular-associated deposition in GCL, IPL, INL, OPL, and choroid in rodents	[47, 107, 117, 139]
	Rat	TgF344-AD		
Tau-related				
pTau	Drosophila	hTau^n^	Differential cell type-specific pTau variants in fly eye; Increased in most retinal layers, strongest in GCL, none in NFL, RPE; NFL, GCL, and AT8^o^ reactivity in *O. degus*	[46, 70, 117, 188]
	Mouse	Tg2576, APP/PS1		
	*O. degus*	Sporadic AD (aged)		
NFT^p^	Mouse	APP/PS1	Immunoreactivity in murine retina, particularly in GCL and RPE	[78, 187, 188]

^a^Drosophila expressing amyloid β-protein precursor

^b^Drosophila expressing β-secretase 1

^c^Model expressing drosophila presenilin-1 protein

^d^Mouse overexpressing APP isoform 695 bearing Swedish double mutations K670M/N671L

^e^Mouse overexpressing APP_SWE_/PS1_ΔE9_ or APP_SWE_/PS1_M146L_

^f^ *Octodon degus*, WT rodent native to Chile showing symptoms of sporadic AD

^g^Glass multiple reporter drosophila model expressing Aβ_42_

^h^Drosophila expressing AβPP-like protein

^i^Outer segment of photoreceptors

^j^Mouse overexpressing APP_SWE_/PS1/TAU_P301L_

^k^Mouse overexpressing APP_SWE/FL/LON_/PS1_M146L/L286V_

^l^Drosophila amyloid β-protein

^m^APP_SWE_/PS1_ΔE9_ rat model

^n^Drosophila expressing human Tau protein

^o^Antibody binding pTau phosphorylated at Ser202 and Thr205

^p^Neurofibrillary tangle

AβPP immunoreactivity has been detected in the eye in a number of AD animal models. These include several Tg strains of drosophila, established Tg mouse models (Tg2576, hTgAPP^tg/tg^, APP_SWE_/PS1_ΔE9_, and APP_SWE_/PS1_M146L/L286V_), and *Octodon degus* (*O. degus*), a WT rodent native to Chile that exhibits symptoms of sporadic AD [8, 46, 47, 56, 117, 139, 188]. Specifically, animal model findings of cytoplasmic AβPP in the photoreceptor layer have been shown to increase in Tg rodents [47, 139], while in *O. degus*, AβPP expression was shown to decrease with age [46]. Strong AβPP and mRNA transcript signals have been reported in the cornea and lens [47, 56]. A drosophila model study has provided additional support for ocular AβPP in AD models, reporting the ubiquitous expression of AβPP in the compound eye [72].

The elevation of soluble and insoluble Aβ peptide levels has been observed in AD animal models, where the increase is age-dependent and corresponds to disease progression. These include the Tg2576, APP/PS1, 3xTg, 5xFAD mice, the TgF344-AD rat, and *O. degus* [1, 46–48, 107, 117, 134, 148, 150, 152, 154, 178, 184]. It has been reported that levels of Aβ_40_ and particularly Aβ_42_ are elevated in the retina, as well as in the lens, vitreous humor, and choroid of AD rodent models (Table 2) [1, 46, 47, 117, 148, 150, 154, 178, 184]. Interestingly, in a study examining the effects of metal in the diet, retinal Aβ abundance was found to increase dramatically in an aluminum-fed 5xFAD mouse [154]. The potential involvement of metal in AD has been reported before [37, 172]. In two other studies using the 5xFAD mouse, retinal Aβ_40_ and Aβ_42_ elevation were reported, with notable detection in the RPE [148, 150]. In the Tg2576 and APP_SWE_/PS1_ΔE9_ mice, possible cytoplasmic Aβ elevation has been documented in the INL, within vacuolar structures in the peripheral GCL, and in the cornea and lens [47]. Interestingly, in this study, enzyme-linked immunosorbent assay (ELISA), other biochemical assays, and immunohistochemistry (IHC) yielded very different results regarding the presence of Aβ peptide in the retina. The AD biomarker was not successfully detected by every method, but definitive evidence was ultimately provided by IHC and ELISA [47]. Two additional studies examining drosophila models of AD have also provided support for the elevation of ocular Aβ peptides [30, 52]. The diverse findings in the studies above emphasize how varied methodologies have driven controversy regarding the detection of AD hallmarks in the eye.

Deposits of insoluble Aβ species and subsequent plaque formation have been documented in the retinas of Tg2576, APP_SWE_/PS1_ΔE9_, APP_SWE_/PS1_M146L/L286V_, 3xTg, and 5xFAD mice, as well as *O. degus* [1, 46–48, 107, 108, 117, 134, 139, 152, 184, 187, 188]. In the Tg2576 mouse, plaques have been identified most consistently in retinal layers ranging from the GCL to the ONL, and rarely in the photoreceptors and optic nerve [117, 184]. In both the APP_SWE_/PS1_ΔE9_ mouse and Tg344F-AD rat models, which share the same double transgenes, plaques and extracellular deposits have been identified in retinal layers ranging from the NFL to the INL, and even in the sclera and choroid [107, 139, 152, 178]. Importantly, Aβ plaques were detected in the retina of APP_SWE_/PS1_ΔE9_ mice as early as 2.5 months of age, 2–3 months prior to their cerebral counterparts [107]. Another study compared plaque load between male and female mice, and found that in old mice, between 12 and 16 months of age, a significantly greater number of female APP_SWE_/PS1_ΔE9_ mice exhibited retinal plaque formation compared with age-matched males [152]. In *O. degus*, a natural model of sporadic AD, Aβ deposits have been observed in the NFL, GCL, and photoreceptors of young animals, while aged animals show intense Aβ staining throughout all retinal layers [46, 86]. The study also reported that the staining of deposits and oligomeric Aβ occurred most intensely in the central retina. In addition, the group noted that while Aβ deposits were confirmed by other means, Congo red did not provide an accurate detection of Aβ [46]. Additional evidence for ocular Aβ deposits and senile plaques has been documented in studies using AD drosophila models [30, 72]. Aβ specifically deposited in and around ocular vasculature, sometimes in association with damaged capillaries, has been detected in the retinas and choroids of APP_SWE_/PS1_ΔE9_ mice and Tg344F-AD rats [47, 117, 139, 178]. Despite a majority agreement on Aβ elevation in the eyes of AD animal models, one study using animal models positive for cerebral plaques was unable to detect Aβ plaques in the eye [47].

Evidence of pTau has been observed from the GCL to the ONL in the Tg2576 mouse and in the soma of RGCs in the APP_SWE_/PS1_ΔE9_ mouse (Table 2) [117, 187]. In *O. degus*, pTau expression has been reported primarily in the NFL and GCL [46]. In the UAS-Gal4 drosophila model of AD, Tau species at various degrees of phosphorylation have been detected in the retina [70]. In addition to pTau species, NFTs have also been detected in the retinas of APP_SWE_/PS1_M146L/L286V_ mice [78, 188].

## Non-specific ocular abnormalities in animal models of AD

Additional ocular changes have been reported in AD animal models, including retinal degeneration, inflammation, structural alterations, and other changes that may be associated with Aβ elevation and deposit formation. Many of these findings mirror those recorded in AD patients (Table 3) [5, 6, 30, 39, 48, 52, 56, 72, 117, 123, 139, 148, 152, 154, 178, 184].

**Table 3:** Non-specific ocular abnormalities in animal models of AD

Abnormality	Species	Model	Findings	References
Retinal degeneration	Drosophila	AβPP^a^/dBACE^b^/dPsn^c^, pGMR-Aβ_42_^d^, dBACE/AβPPL^e^, hAβ_42_tg^f^	Photoreceptor, optic nerve axonal degeneration, and overall abnormality in the fly eye; Thinning, amacrine cell apoptosis and other signs of degeneration in all layers, particularly NFL, GCL, IPL, and RPE	[30, 39, 52, 56, 61, 72, 75, 139, 148, 184, 187]
	Mouse	Tg2576^g^, APP/PS1 ^h^, 5xFAD^i^, hAβPPtg/tg^j^		
Inflammation	Mouse	Tg2576, APP/PS1, 3xTg^k^, 5xFAD, ApoE4 ^l^	Increased microgliosis, MCP-1^m^ upregulation, infiltration by lymphocytes and monocytes, and abnormally shaped GFAP-astrocytes^n^ engulfing Aβ, other signs of inflammation in most retinal layers, particularly in and near GCL, choroid	[6, 48, 61, 117, 139, 152, 154, 178, 187]
	Rat	Tg344-AD^o^		
Structural	Mouse	Tg2576, APP/PS1, 5xFAD, ApoE4	Retina. Synaptic impairment and density decrease of axons and synapses; tight junction attenuation; increased vascular branching and budding; RPE-adjacent Drusen-like deposits along Bruch’s membrane; RGC dendritic complexity, field area, length decreases; cell loss and sparse distribution, GCL displaced cholinergic amacrine cell degeneration, ILM thickening	[5, 6, 62, 123, 148, 184]
	*O. degus* ^p^	Sporadic AD (aged)	Retina. Post-synaptic dysfunction	[8]
	Rat	TgF344-AD	Choroid. Thickness reduction and vascular changes	[178]
	Drosophila	hAβ_42_tg	Lens. Fission resulting in glazed-eye phenotype and reduced compound eye; swelling, organelle disorganization, opacity	[39, 56]
	Mouse	Tg2576, hAβPPtg/tg		
Other	Mouse	Tg2576, APP/PS1, ApoE4	Retina. Double nuclei and hypertrophy in RPE; increased ATP^q^ release; Mitochondrial swelling, crista fragmentation and reduced complexity	[75, 151, 152, 178, 184]
	Rat	TgF344-AD		
	Mouse	ApoE4	Choroid. Increased neovascularization post-LDI^r^, decreased VEGF^s^, damage to neonatal vascular branching	[6, 123]

^a^Drosophila expressing amyloid β-protein precursor

^b^Drosophila expressing β-secretase 1

^c^Model expressing drosophila presenilin-1 protein

^d^Glass multiple reporter drosophila model expressing Aβ_42_

^e^Drosophila expressing AβPP-like protein

^f^Drosophila model expressing human Aβ_42_

^g^Mouse overexpressing APP isoform 695 bearing Swedish double mutations K670 M/N671L

^h^Mouse overexpressing APP_SWE_/PS1_ΔE9_ or APP_SWE_/PS1_M146L_

^i^Mouse overexpressing APP_SWE/FL/LON_/PS1_M146L/L286V_

^j^Drosophila model expressing human AβPP

^k^Mouse overexpressing APP_SWE_/PS1/TAU_P301L_

^l^Apoliprotein E mouse model

^m^Monocyte chemoattractant protein-1

^n^Astrocytes positive for glial fibrillary acidic protein

^o^APP_SWE_/PS1_ΔE9_ rat model

^p^ *Octodon degus*, WT rodent native to Chile showing symptoms of sporadic AD

^q^Adenosine triphosphate, in APP_SWE_/PS1_ΔE9_ model overexpressing ATP

^r^Laser-driven injury

^s^Vascular endothelial growth factor

A number of studies have reported that retinal tissues in rodent models presenting the elevation of the neurotoxic Aβ peptide also show significant degeneration compared with those of control animals [56, 148, 178, 187]. An array of degenerative markers has been documented in different animal models. Cellular swelling, nuclear disorganization, shape irregularity, and organelle loss in cortical fiber cells have been observed in the lens of an hTgAPP^tg/tg^ mouse [56]. The RPE has shown hypopigmentation, large vacuoles, and Bruch's membrane thickening with drusen-like deposits in a 5xFAD mouse [148], as well as hypertrophy in the TgF344-AD rat [178]. RGC distribution in the retina of APP_SWE_/PS1_ΔE9_ mice is sparse and overall numbers show a significant decrease when compared with matched WT controls [61, 75]. Amacrine cell apoptosis has also been noted in the retina of the APP_SWE_/PS1_ΔE9_ mouse [61]. Evidence from drosophila studies supportive of ocular degeneration in AD models has indicated severe photoreceptor abnormality, lens fission, and axonal degeneration in the optic nerve [30, 39, 52, 72].

Inflammatory processes are well documented in the brains of AD patients [68, 128, 186]. In 2008, Ning and colleagues were the first to show that the accumulation of Aβ in the retina of Tg mice was associated with neurodegeneration and inflammation [139]. Since then, several reports of ocular inflammation in rodent models of AD have indicated increases in microgliosis, GFAP^+^ astrogliosis, retinal infiltration of lymphocytes and monocytes, and upregulation of MCP-1, among other markers in many layers of the retina and choroid [6, 48, 61, 117, 139, 152, 154, 178, 187].

Structural changes in the retinas of AD animal models have included tight junction attenuation, variations in vascular branching and budding, and decreases in complexity, field area, and length of RGC dendrites (Table 3) [5, 6, 123, 148, 184]. While synaptic density in the Tg2576 mouse has shown no change in either pre- or post-synaptic markers [184], synaptic loss and impairment, typically related to cognitive deficits when observed in the brain, have been reported in the ApoE4 mouse retina [5, 6]. Changes observed outside of ocular CNS tissues in rodent and fly eyes have included thinning of the choroid [178], as well as cellular swelling, organelle disorganization, and opacity in the lens [39, 56]. Furthermore, altered expression levels of various proteins and mRNA transcripts essential to normal cell function have been detected, including a report of increased ATP release in the retina of an APP/PS1 mouse model [151].

Intracellular malformations have also been documented in retinal cells, including increases in double nuclei and hypertrophy in the RPE, as well as cellular swelling, crista fragmentation, and complexity reduction in mitochondria of Tg rodent models [152, 178, 184]. In addition, two studies investigating ocular changes in the ApoE4 mouse reported increased neovascularization following laser-driven injury and decreased VEGF levels in the choroid [6, 123]. Overall, the above studies, predominantly those pertaining to genetic murine models of AD, indicate that the effects of Alzheimer’s known to afflict the brain, especially synaptic loss and neuronal degeneration, also manifest in the retina.

## In vivo imaging of AD in the eyes of patients and animal models

Visualization of the retina and its AD-related abnormalities may be achieved with non-invasive optical imaging technologies and advanced electroretinogram techniques. Advanced OCT has been widely used in recent years to accurately image cross sections of the retina. This technique has provided evidence for significant thinning of the peripapillary NFL, macular volume loss, and nerve fiber density decrease in patients with mild to severe AD, suggesting that thinning might occur early in disease progression [10, 14, 50, 62, 87, 91, 92, 100, 104, 113, 114, 121, 135, 142, 145]. Used in conjunction with Fundus Auto-Fluorescence (FAF), a method for detection of highly fluorescent structures, areas of interest for OCT examination have been suggested, thereby leading to a possible visualization of perimacular and perivascular Aβ deposits primarily in the OPL, GCL, and NFL of AD patients [100]. A modified HRA OCT system has been further employed to detect pTau in the GCL, OPL, IPL, and INL of a P301S mouse model [163].

Thus far, a consensus has not been met regarding the correlation between NFL thinning and degree of cognitive impairment. One study that measured impairment by MMSE and Montreal Cognitive Assessment (MOCA) found a positive correlation [142], while two others specifically reported no correlation between NFL thinning and either MMSE score or AD status [62, 111]. Notably, a study investigating NFL thinning as a diagnostic tool reported successful prediction of AD status from NFL thickness data [114].

While ex vivo staining has suggested that amyloid plaques can be detected in the eye, the first in vivo detection of Aβ deposits in a Tg model of AD came in 2010, when Koronyo-Hamaoui et al. reported high-resolution detection of curcumin-labeled Aβ plaques in the APP_SWE_/PS1_ΔE9_ retina using an adapted optical imaging microscope [107, 108]. Other improvements and modifications to established OCT techniques, including Fourier Domain OCT, Spectral Domain OCT, Functional OCT, and Doppler OCT, have allowed researchers to document degeneration in specific ocular tissues and cell types, as well as changes in blood flow and blood oxygen saturation in the retina [17, 106, 110, 114, 124, 181].

Various imaging techniques have been used to study other aspects of degeneration in the AD eye. For example, lens opacity measured by light scattering was not found to correlate significantly with AD disease progression [18]. Digital photography has been used to examine changes to retinal vasculature, including vascular narrowing and attenuation, changes in tortuosity, increased width variation, and reduction of branching complexity [32, 59]. Fluorescent Ligand Eye Scanning (FLES) is another approach to detect Aβ peptides in AD lenses in vivo, which was used in another study to predict clinical diagnoses in probable AD patients [101, 102]. An additional study examining changes in retinal blood oxygen saturation used spectrophotometric non-invasive retinal oximetry to report the elevation of blood oxygen saturation, yielding data similar to that of AMD in AD patient retinas [49]. Furthermore, scanning with laser ophthalmoscopy (SLO) has revealed a reduction in the number of fibers in the AD optic nerve [40]. Confocal SLO has also been used for in vivo monitoring of apoptotic RGC death in a 3xTg AD mouse model, which could be applied to future research on neurodegeneration in AD patients [35]. Dysfunction in RGCs and changes in the optic nerve were also detected by pERG in patients with AD [109, 146, 147].

Although many of these changes are common to other neurodegenerative diseases [120], the ability to monitor increasingly detailed changes in the AD eye can illuminate those processes specific to the disease, such as deposition of Aβ. More importantly, these studies suggest that retinal imaging technologies with high resolution and sensitivity could be adapted to detect AD-specific pathology, which could facilitate the early diagnosis and monitoring of disease progression.

## Therapeutic response in the retina of murine AD models

Advances in imaging of retinal Aβ in vivo facilitate the possibility of monitoring changes in amyloid burden in response to therapeutic intervention. Immunotherapies targeting Aβ deposits and accumulation have been studied in a handful of rodent models.

The first examination of the effects of immunization therapy on AD-related pathology in the rodent eye came from Liu et al. [117]. In this study, experimental groups of Tg2576 mice were immunized subcutaneously with a number of Aβ-related molecules [i.e., islet amyloid polypeptide (IAPP), Aβ oligomer, or Aβ fibril], and various parameters were measured. Instances of retinal plaque formation and resulting plaque density observed by immunohistochemistry were shown to decrease, though these results reached statistical significance solely in the Aβ oligomer- and IAPP-immunized groups. Amyloid angiopathy score increased significantly in all groups. Interestingly, retinal thinning was attenuated, but microglial infiltration and astrogliosis increased in the immunized groups compared with controls [117].

Among the considerations involved in developing new therapies for neurodegeneration is the possibility of repurposing existing or even FDA-approved drugs to combat different pathologies. In 2012, Koronyo et al. published findings from a study using the sub-cutaneous immunization of glatiramer acetate (GA), a drug approved for the treatment of relapsing-remitting multiple sclerosis [108, 164]. Their previous study also involved a modified myelin oligodendrocyte glycoprotein-derived peptide (MOG45D) loaded on dendritic cells (DC-45D) in the retinas of APP_SWE_/PS1_ΔE9_ mice [107]. In the latter study, the authors measured and described the ex vivo quantitative reduction of amyloid plaque burden in the brain matching that observed in the retina [107]. In the subsequent study, in vivo imaging of mouse retinas following GA immunization displayed a reduction in Aβ plaque number after 1 month, and further reduction, as well as a dynamic pattern of plaque formation and clearance, after 2 months [108]. Next, Yang et al. investigated the effects of bone marrow transplantation (BMT) on APP_SWE_/PS1_ΔE9_ mice, and reported a significant reduction in numbers of both retinal and cerebral Aβ deposits in BMT-treated Tg mice. The study also reported that the total number of retinal microglia, possibly involved in phagocytosis of Aβ plaques, was normalized to non-disease levels comparable to those seen in WT mouse retinas. GCL neuronal loss, inner retinal thinning, and other signs of age-related degeneration were mitigated in BMT-treated mice [187].

More recent investigations have yielded other promising results. He et al. [78] reported that treatment with Ginsenoside Rg1 significantly decreased NFT formation in the RPE cells of APP_SWE_/PS1_M146L_ mice compared with untreated Tg controls. Parthasarathy et al. [150] investigated the effects of intra-vitreally delivered sNEP (a recombinant form of the neprylisin catalytic domain) in the eyes of 5xFAD mice. The study reported a decrease in Aβ levels in sNEP-treated eyes compared with untreated Tg controls [150]. Retinal Aβ_40_ appeared to have been more strongly affected, as a significant reduction of Aβ_40_ was observed 2 h following treatment, while the reduction of Aβ_42_ reached statistical significance only after 3 days. Although ERG response improved with sNEP treatment, this finding was not statistically significant [150]. Recently, Gao et al. [61] reported that memantine (MEM), an uncompetitive antagonist of the *N*-methyl-d-aspartate receptor, markedly increased RGC count (by NeuN-IR staining) and significantly decreased the number of apoptotic RGCs in retinas of MEM-treated Tg mice when compared with untreated Tg controls. Müller cell adapted response appeared to be inhibited in MEM-treated mice, and inner limiting membrane (ILM) thickening was mitigated compared to that of the untreated Tg controls. In addition, visual function showed improvement in MEM-treated mice, as the pERG P50 component showed a significant increase in amplitude and the pERG P2 component delay was significantly attenuated compared with deficits observed in untreated Tg controls [61].

Although these animal model studies involve diverse methodology, they collectively suggest that the retina is a tissue that may faithfully mirror brain pathology. Furthermore, the availability of the retina as a site for clinical monitoring of disease progression in response to therapeutic intervention also alludes to the possibility of potential therapeutic intervention via the retina. In the current absence of effective therapy for AD, the retina may yet prove useful as a site of retrograde drug administration, by exploiting the molecular delivery systems of the optic nerve.

## Conclusions

Over the past decade, our understanding of AD has grown far beyond its established definitive signs, cerebral Aβ plaques and NFTs. In this highly dynamic field, novel disease biomarkers are continually revealed. Amyloid-related findings include phosphorylated or conformationally diverse forms of Aβ, prone to extra- and intracellular aggregation. Other key molecular findings include assemblies of pTau, which are typically intracellular, but can also be observed in extracellular space, and may exhibit self-propagating properties. Many of these hallmarks, along with neuroinflammation and related abnormalities, have been observed in the retinas of AD patients and animal models.

Owing to the embryological ties of the neuroretina and brain structures affected by AD, it is no surprise that research has yielded increasing indications of degeneration in the AD retina as well. Recent evidence of early Aβ aggregation and amyloid-related neuronal degeneration in retinal tissues has mirrored that reported in human AD and Tg animal model brains. This not only supports the status of the retina as a target of presymptomatic AD imaging, but also suggests that Alzheimer’s simultaneously affects both the brain and the retina.

Advances in retinal imaging and evidence of a positive response to therapy in the eyes of AD animal models hold promise for widespread population screening, early diagnosis and monitoring, and ultimately developing disease-modifying intervention. Although we learn much from observing AD in the brain, a key goal is to screen for the earliest signs and progression of the disease, and to intervene before it manifests as irreversible clinical symptoms. Therefore, one can no longer ignore the possibility that the retina—a CNS tissue uniquely accessible for direct, high resolution, non-invasive imaging—provides invaluable access to study and monitor Alzheimer’s disease.
